# A case–control study of prevalence of anemia among patients with type 2 diabetes

**DOI:** 10.1186/s13256-016-0889-4

**Published:** 2016-05-04

**Authors:** Samuel Antwi-Bafour, Samuel Hammond, Jonathan Kofi Adjei, Ransford Kyeremeh, Alexander Martin-Odoom, Ivy Ekem

**Affiliations:** 1 Department of Medical Laboratory Sciences, School of Biomedical and Allied Health Sciences, College of Health Sciences, University of Ghana, P. O. Box KB 143, Korle-Bu Accra, Ghana; 2 Medical Affairs Directorate, Korle-bu Teaching Hospital, Accra, Ghana

**Keywords:** Hemoglobin concentration, Anemia, Renal insufficiency, Diabetes

## Abstract

**Background:**

Anemia is defined as a reduction in the hemoglobin concentration of blood, which consequently reduces the oxygen-carrying capacity of red blood cells such that they are unable to meet the body’s physiological needs. Several reports have indicated that anemia mostly occurs in patients with diabetes with renal insufficiency while limited studies have reported the incidence of anemia in people with diabetes prior to evidence of renal impairment. Other studies have also identified anemia as a risk factor for the need for renal replacement therapy in diabetes. Understanding the pathogenesis of anemia associated with diabetes may lead to the development of interventions to optimize outcomes in these patients. The aim of this study was therefore to determine the prevalence of anemia among patients with type 2 diabetes.

**Methods:**

A total of 100 (50 with type 2 diabetes and 50 controls) participants were recruited for our study. Participants’ blood samples were analyzed for fasting blood glucose, full blood count and renal function tests among others. The prevalence of anemia was then determined statistically.

**Results:**

A high incidence of anemia was observed in the cases. Of the patients with diabetes, 84.8 % had a hemoglobin concentration that was significantly less (males 11.16±1.83 and females 10.41±1.49) than the controls (males 14.25±1.78 and females 12.53±1.14). Renal insufficiency determined by serum creatinine level of >1.5 mg/dL, estimated glomerular filtration rate <60 ml/minute/1.73 m^2^, and erythropoietin levels was also observed to be high in the cases (54.0 %; with mean creatinine concentration of 3.43±1.73 and erythropoietin 6.35±1.28 mIU/mL). A significantly increased fasting blood glucose, urea, sodium, potassium, and calcium ions were observed in the cases (7.99±1.30, 5.19±1.99, 140.90±6.98, 4.86±0.53 and 1.47±0.31 respectively) as compared to the controls (4.66±0.54, 3.56±2.11, 135.51±6.84, 4.40±0.58 and 1.28±0.26 respectively). Finally, a significant association between hemoglobin concentration and fasting blood glucose was also observed in the cases.

**Conclusions:**

The findings suggest that a high incidence of anemia is likely to occur in patients with poorly controlled diabetes and in patients with diabetes and renal insufficiency.

## Background

Deficiency in the oxygen-carrying capacity of blood due to a diminished erythrocyte mass or reduction in the hemoglobin (Hb) concentration of the blood may indicate anemia [1]. This leads to the blood not being able to meet the body’s physiological needs. It is caused by either an excessive destruction or diminished production of red blood cells [2]. Anemia is associated with increased perinatal mortality, child morbidity and mortality, impaired mental development, immune incompetence, increased susceptibility to lead poisoning, and decreased performance at work [3].

Anemia has a high prevalence and is considered a public health problem affecting developing and developed countries. It occurs at all stages of life, especially in pregnant women and children [4]. Globally, 1.62 billion people are anemic, corresponding to 24.8 % of the global population. The highest prevalence of 47.4 % is in preschool-age children while the lowest prevalence of 12.7 % is in men [5]. Data from World Health Organization (WHO) regional estimates, which were generated for preschool-age children, pregnant women, and non-pregnant women, indicated that the highest proportion of people affected are in Africa (47.5 to 67.6 %), while the greatest number affected are in Southeast Asia where 315 million individuals in the three population groups are affected [5]. In Ghana, anemia was ranked as the fourth leading reason for hospital admissions and the second factor contributing to death after a review of the disease profile and pathology reports of selected hospitals [4].

Diabetes mellitus (DM) is a non-infectious disease that also has a high prevalence worldwide [6]. It is a carbohydrate metabolism disorder which results in hyperglycemia due to either absolute insulin deficiency or reduced tissue response to insulin or both [6]. Diabetes, especially when poorly controlled, leads to complications such as nephropathy, retinopathy, and neuropathy as well as several disordered metabolic processes including oxidative stress which causes oxidative damage to tissues and cells [7]. Anemia is one of the commonest blood disorders seen in patients with diabetes [8]. Many research studies have reported that anemia mostly occurs in patients with diabetes who also have renal insufficiency [9]. A few other studies have also reported an incidence of anemia in diabetics prior to evidence of renal impairment [10]. Anemia occurs earlier and at a greater degree in patients presenting with diabetic nephropathy than those presenting with other causes of renal failure [10].

Developing countries including Ghana carry the most significant proportion of the reported cases of anemia whose etiology is often multifactorial. The main causes of anemia may include: dietary iron deficiency; infectious diseases such as malaria, hookworm, and schistosomiasis; micronutrient deficiencies including folate, vitamin B_12_ and vitamin A; or inherited conditions that affect red blood cells such as thalassemia and sickle cell disease [11]. Again, people with chronic illnesses such as kidney problems, cancer, diabetes, and related conditions are at a higher risk for developing anemia. However, the most significant contributor to the onset of anemia is iron deficiency which has often been used as synonymous to anemia, and the prevalence of anemia used as a proxy for it [12].

Of the cases of anemia, 50 % are therefore attributable to iron deficiency [13], but this proportion could vary among population groups in different areas according to local conditions. Low dietary intake of iron, poor absorption of iron from diets high in phytate or phenolic compounds, and period of life when iron requirements are especially high (that is, growth and pregnancy) are the main risk factors of iron deficiency anemia [12]. Blood loss resulting from menstruation and parasitic infestation such as hookworm, *Ascaris*, and schistosomiasis also contribute to the lowering of Hb concentration resulting in anemia. Malaria, cancer, tuberculosis, and HIV can also contribute to the burden of anemia. An increase in the risk of anemia could also result from deficiencies in copper and riboflavin. The impact of hemoglobinopathies on anemia prevalence also needs to be considered within some populations [12]. The different causes of anemia may work in concert, so in a single individual, various nutrient deficiencies, chronic illnesses and different infestations may all play a role [14]. It therefore remains important to establish in various populations the role that different causative factors play in the overall alarming prevalence of anemia [15].

Vitamin B_12_ deficiency and folic acid deficiency lead to megaloblastic anemia that results from inhibition of DNA synthesis during red blood cell production [16]. The mechanism of this phenomenon is loss of B_12_-dependent folate recycling, followed by folate-deficiency loss of nucleic acid synthesis, leading to defects in DNA synthesis. Vitamin B_12_ deficiency alone in the presence of sufficient folate will not cause the syndrome. Megaloblastic anemia that is not due to hypovitaminosis may be caused by antimetabolites that poison DNA production directly, such as some chemotherapeutic or antimicrobial agents (azathioprine or trimethoprim) [17]. The pathological state of megaloblastosis is characterized by many large immature and dysfunctional red blood cells (megaloblasts) in the bone marrow and by hypersegmented neutrophils [18].

Anemia is also the most frequent hematological manifestation in patients with malignant diseases [19]. It may be the first diagnostic clue to an underlying malignant disease and may contribute to the patient’s symptoms from the disease as well as affect treatment decisions. It is known that tumor-associated cytokine production is a major factor in the anemia of malignancy. In fact, numerous in vitro studies have illustrated the central role of tumor necrosis factor-alpha (TNF-α) in the pathogenesis of anemia [20]. Other cytokines, such as interleukin-6 (IL-6), IL-1 and interferon-γ, have also been shown to inhibit erythroid precursors in vitro, albeit to a lesser extent [21]. The duration and severity of anemia seems to be related to the type of cancer, extent of disease, and myelosuppressive potency of chemotherapy [22]. The most common form of anemia seen in patients with cancer or hematological malignancies results from the underproduction of red cells: a hypoproliferative anemia characterized by a low reticulocyte production index and the absence of marrow erythroid hyperplasia despite significant persistent anemia. Again, one of the hallmarks of malignancy-associated anemia is the reduction in endogenous erythropoietin (EPO) levels with respect to the degree of anemia [23].

Malignancy can affect bone marrow as well (bone marrow fibrosis) and this can also result in anemia [24]. Bone marrow has a rich blood supply and is therefore a common site for a metastasis to develop [24]. Cancers of the breast, prostate, and lung are the commonest type to do this although almost all cancers have this capability. Once in the marrow the cancer cells can multiply easily and the tumor deposit enlarges, occupying more and more of the marrow space, so reducing the amount of blood-producing marrow and the subsequent anemia [25]. There are some tumors that arise from the bone marrow tissue itself, such as some types of leukemia and multiple myeloma. As these are more directly involved with the bone marrow’s function they are more commonly associated with anemia.

A normocytic normochromic anemia is also common in patients with a variety of inflammatory disorders and there are many contributing factors [26]. In inflammation, from whatever cause, IL-6 induces the liver to produce hepcidin. Hepcidin decreases iron absorption from the bowel and blocks iron utilization in the bone marrow. Iron may be abundant in the bone marrow, but is not absorbed and is not in the circulation, and so is not available for erythropoiesis. Again, some chemotherapeutic agents induce anemia by impairing hematopoiesis [27]. In addition, nephrotoxic effects of particular cytotoxic agents, such as platinum salts, can also lead to the persistence of anemia through reduced EPO production by the kidney [28]. The myelosuppressive effect of cytotoxic agents might accumulate over the course of chemotherapy. This results in a steady increase in the incidence of anemia with every new cycle of chemotherapy.

Furthermore, many diseases, conditions, and factors can cause our body to destroy its red blood cells leading to hemolytic anemia [29]. These causes can be inherited or acquired but sometimes the cause is not known. Hemolytic anemia can lead to many health problems, such as fatigue, pain, arrhythmias, and heart failure [30]. There are many types of hemolytic anemias and treatment and outlook depend on what type and how severe it is. The condition can develop suddenly or slowly and symptoms can range from mild to severe. Hemolytic anemia often can be successfully treated or controlled. Mild hemolytic anemia may need no treatment whereas severe hemolytic anemia will require prompt and proper treatment, or it may be fatal. Inherited forms of hemolytic anemia are lifelong conditions that may require ongoing treatment but acquired forms may go away if the cause of the condition is found and corrected [31]. The following are some examples of diseases that lead to hemolytic anemia: sickle cell disease, thalassemias, hereditary spherocytosis, glucose-6-phosphate dehydrogenase (G6PD) deficiency, pyruvate kinase deficiency, acquired hemolytic anemias, immune hemolytic anemia, and mechanical hemolytic anemias [32–34].

Results from a study by Rossing *et al.* showed a significant association between a lower Hb concentration and a decline in glomerular filtration rate (GFR) [35]. Other recent studies have also identified anemia as a risk factor for the need for renal replacement therapy in diabetes [36]. Furthermore, anemia has a negative impact on the survival of patients with diabetes and is considered to be an important cardiovascular risk factor associated with diabetes and renal disease [35, 36]. There is therefore a need for more studies on the incidence and prevalence of anemia among patients with diabetes particularly those with renal malfunction. This study therefore aimed to determine the prevalence of anemia due to renal insufficiency among patients with type 2 diabetes and the outcome showed a high incidence of anemia among them. The findings suggest that anemia is likely to occur in patients with diabetes with renal insufficiency, particularly when it is poorly controlled. It is therefore believed that presentation of the outcome will help increase the level of awareness and understanding of anemia among patients with diabetes, which will eventually lead to the development of interventions to optimize treatment outcomes in them.

## Results

### Participants’ demographics

The study included 100 participants, consisting of 50 participants with diabetes (15 males/35 females) and 50 participants without diabetes (14 males/36 females) who consented to participate in the study. Mean ages recorded for cases and controls were 55.62±10.37 years and 44.11±15.30 years respectively (Table 1). The medical records of the participants were examined and they were taken through a physical examination for signs and symptoms of anemia. However, none of them showed any signs of anemia, possibly due to the fact that there may be no symptoms in some people who have anemia. Other diseases such as cancer and myelodysplasia as well as other causes of anemia as discussed in the introduction were ruled out among the study group.

**Table 1:** Summary of demographic characteristics of participants with diabetes (cases) and controls

Parameters	Cases	Controls
*n*	50	50
Male/female	15/35	14/36
Age (years)	55.62±10.37	44.11±15.30

Table 2 shows the hematological parameters of the blood samples we analyzed that were obtained from the study participants. Hb concentration was observed to be significantly decreased in the cases as compared to the controls. Ferritin and total iron-binding capacity (TIBC) levels were found to be normal in most of the cases and lower in the control participants who were found to be anemic. The mean cell volumes (MCVs) were higher in the cases than the controls.

**Table 2:** Hematological parameters in participants with diabetes (cases) and controls

Parameters		Cases	Controls	*p* value
Hb	Male (13.5–17.5 g/dL)	11.16±1.83	14.25±1.78	0.000*
	Female (12.0–16.0 g/dL)	10.41±1.49	12.53±1.14	0.000*
Ferritin	Male (20–200 μg/L)	83.66±1.19	91.96±1.15	0.000*
	Female (20–120 μg/L)	50.40±1.10	55.40±1.29	0.000*
MCV (80–95 fL)		86.19±1.09	84.02±1.11	0.000*
TIBC (255–450 μg/dl)		315.30±2.68	360.10±2.84	0.001*

*Hb* hemoglobin, *MCV* mean cell volume, *TIBC* total iron-binding capacity (*p value <0.05 was considered significant)

Table 3 shows the biochemical parameters of the blood samples we analyzed that were obtained from the study participants. A significant increase in fasting blood glucose (FBG) concentration was observed in the cases compared to controls (*p*=0.000). Significant increases in urea, sodium (Na), potassium (K), and calcium (Ca) concentrations were also observed in the cases as compared to controls. Creatinine concentrations were almost similar in both cases and controls although they were on the high side. EPO and estimated glomerular filtration rate (eGFR) levels were lower in cases than in controls. Glycated Hb (HbA1c) levels were also found to be higher in cases (particularly those with anemia) than in controls.

**Table 3:** Biochemical parameters in participants with diabetes (cases) and controls

Parameters		Cases	Controls	*p* value
FBG (3.8–6.1 mmol/L)		7.99±1.30	4.66±0.54	0.000*
Erythropoietin (4.1–19.5 mIU/mL)		6.35±1.28	12.82±1.99	0.000*
eGFR	Female (80–110 ml/minute/1.73 m^2^)	85.41±1.49	87.53±1.14	0.000*
	Male (90–120 ml/minute/1.73 m^2^)	90.16±1.83	94.25±1.78	0.000*
Urea (7–18 mg/dL)		5.19±1.99	3.56±2.11	0.000*
Na (135–145 mmol/L)		140.90±6.98	135.51±6.84	0.000*
K (3.5–5.0 mmol/L)		4.86±0.53	4.40±0.58	0.000*
Cl (95–105 mmol/L)		105.30±3.95	103.40±3.65	0.000*
Ca (2.1–2.8 mmol/L)		1.47±0.31	1.28±0.26	0.001*
Creatinine (0.6–1.5 mg/dL)		2.35±1.74	2.37±1.35	0.945
HbA1c (4–7 %, 7–8 %, ≥8.5 %)		7.80±1.04	4.61±1.94	0.001*

*Ca* calcium, *Cl* chloride, *eGFR* estimated glomerular filtration rate, *FBG* fasting blood glucose, *HbA1C* glycated hemoglobin, *K* potassium, *Na* sodium (*p value <0.05 was considered significant)

From Table 4, it was seen that participants with diabetes had a high incidence of anemia in both males and females (86.7 % and 82.9 % respectively) and 19.4 % of the females in the control group were also anemic. Anemia was defined by an Hb <13.0 g/dL in men and Hb <12.0 g/dL in women [20]. Three types of anemia were seen morphologically and by the MCVs obtained: hypochromic microcytic (MCV <80 fL), normochromic normocytic (MCV 80–95 fL), and normochromic macrocytic (MCV>95 fL).

**Table 4:** Incidence of anemia in participants with diabetes (cases) and controls according to gender

Cases	Anemic (%)	Non-anemic (%)
Male	13 (86.7)	2 (13.3)
Female	29 (82.9)	6 (17.1)
Controls		
Male	1 (7.1)	13 (92.9)
Female	7 (19.4)	29 (80.6)

Renal insufficiency was determined by serum creatinine level >1.5 mg/dL and eGFR <60 ml/minute/1.73 m^2^. A high incidence of renal insufficiency (54.0 %) was observed in the participants with diabetes compared to controls (Table 5). Out of the 42 (84 %) cases who were anemic, 31 (73.8 %) showed low eGFR, which is an indication of renal insufficiency, with the remaining 11 (26.2 %) having higher eGFR and therefore normal renal function. Among the controls, 9 (18 %) were found to be anemic and 7 (14 %) had high eGFR while the remaining 2 (4 %) had low eGFRs.

**Table 5:** Incidence of renal insufficiency in participants with diabetes and controls

	Present (%)	Absent (%)
Participants with diabetes	27 (54.0)	23 (46.0)
Mean	3.43±1.73	1.07±0.28
Controls	13 (26.0)	37 (74.0)
Mean	3.13±1.15	0.99±0.23

**Fig. 1:**
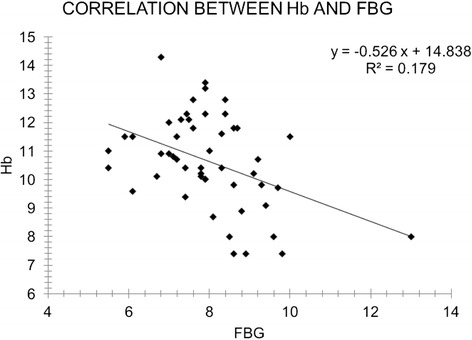
Correlation between hemoglobin concentration and fasting blood glucose. *FBG* fasting blood glucose, *Hb* hemoglobin

A positive correlation was seen between the degree of anemia and HbA1c in patients with diabetes, supporting the hypothesis that there is a higher incidence of anemia among poorly controlled diabetics. Also a negative correlation was observed between Hb and hyperglycemia (FBG) in the diabetic population according to gender (Fig. 1). However, only the female population’s correlation was significant (Table 6).

**Table 6:** Correlation between hemoglobin and hyperglycemia

Pearson’s correlations	*R*	*p* value
Hb and HG (female)	–0.465*	0.005*
Hb and HG (male)	–0.382	0.159

*Hb* hemoglobin, *HG* hyperglycemia, *correlation is significant at *p* value <0.05 (two-tailed)

## Discussion

Anemia is defined as a low level of Hb in the blood and evidenced by fewer numbers of functioning red blood cells. The WHO considers men with a Hb concentration <13.0 g/dL or packed cell volume (PCV) <39 % anemic and women with Hb <12.0 g/dL or PCV <36 % to be anemic [37]. Data from our study show a high incidence of anemia (86.7 % in males; 82.9 % in females) in participants with diabetes, predicting the necessity to assess patients with diabetes for anemia during diagnosis and management. HbA1c was found to be positively correlated whereas FBG was found to be negatively correlated with anemia in the participants with diabetes. This suggests that the incidence of anemia is likely to increase in poorly controlled diabetes, and therefore reducing blood glucose levels could help reduce the risk of anemia in diabetic populations.

Out of the 42 (84 %) cases who were anemic, 31 (73.8 %) showed low eGFR, which is an indication that their anemia may be due to renal causes. They subsequently presented with normochromic normocytic anemia. The remaining 11 (26.2 %) had higher eGFRs and possibly normal renal function; they presented with both hypochromic microcytic (8; 19.1 %) and normochromic macrocytic (3; 7.1 %) anemia. These were suspected to be due to iron and B_12_/folate deficiency respectively. Among the controls, nine (18 %) were found to be anemic and here the majority (7; 14 %) had high eGFRs and presumably normal functioning kidneys; they presented with hypochromic microcytic (5; 10 %) and normochromic macrocytic (2; 4 %) anemia. The remaining two (4 %) had low eGFRs and were deemed to have renal insufficiency-related anemia.

A previous study reported a 15.3 % incidence of anemia in participants with diabetes without renal insufficiency [38]. The study added that patients who have poorly controlled diabetes were at greater risk of anemia than those with controlled diabetes. Another study reported that 7.2 % of diabetics with normal renal function had anemia [39]. Again, other studies have reported that 20 % [8] and 19.6 % [39] of participants with diabetes with renal insufficiency had anemia.

Anemia is a key indicator of chronic kidney disease (CKD) but occurs earlier in the course of diabetic kidney disease and may be more severe than previously realized [40, 41]. In patients with diabetes, anemia may be the result of diminished EPO production by the failing kidney. It has been suggested in other studies that this may be due to a reduction in the number of specific EPO-synthesizing interstitial cells and disruption of the interstitial anatomy or vascular architecture [42, 43]. A role has also been suggested for autonomic dysfunction through a relative or absolute imbalance in sympathetic/parasympathetic tone based on the hypothesis that EPO production may be modulated, in part, by the autonomic nervous system [44, 45]. Most patients with diabetes are rarely tested for anemia and are unaware of the link between anemia and kidney disease. A pan-European study was therefore undertaken by Stevens *et al.* (2003) to investigate the level of awareness and understanding of anemia among patients with diabetes [46]. They concluded that anemia has a significant impact on the quality of life of patients with diabetes and although patients are aware of anemia, their awareness of being tested for anemia is low [46].

The estimated prevalence of anemia in people with diabetes depends on essentially arbitrary criteria used to define the presence or absence of anemia. Nonetheless, studies in patients with renal impairment suggest that deleterious effects begin with Hb <11 g/dl, meaning that 7 % of patients with diabetes may benefit from intervention according to current guidelines [47]. Using this definition, nearly one in four patients with diabetes (23 %) may have anemia warranting evaluation. Although other smaller studies have suggested that the prevalence of anemia is increased in diabetes, their surveys have generally selected patients with overt nephropathy [48]. In contrast, the Predialysis Survey on Anemia Management (PRESAM) failed to show a difference between patients with and without diabetes [49].

Again a study by Thomas *et al.* (2003) demonstrated that anemia is an early and common complication of diabetes and patients at greatest risk of anemia can be readily identified [50]. In the study, 60 % of patients with anemia warranting investigation had eGFR <60 ml/minute^−1^/1.73 m^2^ and nearly half (46 %) of the patients with macroalbuminuria had anemia [51, 52]. As the risk of anemia is strongly associated with eGFR in the study by Thomas *et al.*, it seems likely that supplementation with EPO could correct anemia, particularly in the patients with anemia and adequate iron stores [50]. However, potential benefits need to be balanced against the risks of adverse arterial effects and the complications of EPO use, including hypertension and pure red cell aplasia.

The high incidence of anemia observed in our study may be due to the relatively small number of study participants about half of whom presented with renal insufficiency (Table 5). Anemia due to renal insufficiency is primarily as a result of reduced secretion of EPO by the failing kidneys, and anemia subsequently occurs when creatinine clearance is less than 50 mL/minute. This is observed earlier in patients with diabetes with renal insufficiency or disease [10]. The high incidence of anemia may also be due to other risk factors related to DM. Several studies have reported factors that increase the risk of anemia, which include; damage to renal interstitium due to chronic hyperglycemia and consequent formation of advanced glycation end products by increased reactive oxygen species, and systemic inflammation as well as reduced androgen levels induced by diabetes [8, 10]. A limiting factor worthy of mention is our sample size; a larger sample would have increased the power of the study outcome. We also did not determine the HIV status of our study participants and cannot comment on the role of HIV on the prevalence of anemia in this particular study population, although infection with HIV has emerged as an additional risk factor for anemia [53].

## Conclusions

The findings of our study suggest that the high incidence of renal insufficiency observed in the participants with diabetes, among other factors, could have influenced the high incidence of anemic conditions seen. Anemia is therefore likely to occur in poorly controlled diabetes and in patients with diabetes with renal insufficiency. Including routine hematological (Hb) tests in the treatment of diabetes and considering factors such as glycemic control and renal sufficiency among others could help reduce anemia in diabetes and the possible complications that may come with it.

## Methods

### Study design

The study was a case–control study conducted between the months of March and August 2014.

### Materials

Some of the materials used included: ABX Micros 60 Haematology Analyzer, Starlyte V & Human Reader chemistry analyzers, 5 ml K_2_EDTA test tubes, plain test tubes, fluoride tubes, syringes, needles, absolute methanol, and cotton wool.

### Ethical consideration

Ethical clearance for this research in accordance with the 1964 Declaration of Helsinki and its later amendments or comparable ethical standards was sought from the Ethics and Protocol Review Committee of the School of Biomedical and Allied Health Sciences, University of Ghana, Legon (ED ID NO:SAHS-ET./10359975/2013-2014). All the participants gave their informed consent before their samples were collected.

### Specimen collection and processing

From each participant, 6 ml of an overnight fasting venous blood was collected as follows: 2 ml into an ethylenediaminetetraacetate (EDTA) tube for hematological profile, 2 ml into a plain tube for renal function tests (RFTs), and 2 ml into a fluoride tube for FBG and HbA1c analysis.

### Full blood count test

Full blood count comprising red cell count, Hb, white cell count and differentials, platelets as well as Hb indices were determined from the whole blood in the EDTA test tubes using ABX Micros 60 Haematology Analyzer (Horiba-ABX, Montpellier, France). Ferritin and TIBC were also done.

### Renal function test

This was determined using two chemistry analyzers (Starlyte V, USA for the electrolyte measurements; Human Reader, Germany for urea and creatinine measurements). Blood urea nitrogen, EPO, electrolytes, creatinine, and eGFR were done for each participant to assess their renal function.

### FBG estimation

Glucose is oxidized in the presence of glucose oxidase to form glucuronic acid and hydrogen peroxide. Hydrogen peroxide in the presence of peroxidase reacts with 4-aminophenazone and phenol to produce a colored quinone imine complex which is measured against a reagent blank at an absorbance of 550 nm.

The FBG for each participant was estimated from the fluoride tube.

### Statistical analysis

The data obtained were cleaned and entered into Statistical Package for Social Scientists (SPSS) version 20.0. Descriptive statistics such as frequencies and percentages were used to summarize categorical variables such as gender. Continuous variables such as Hb concentration, ferritin concentration, and TIBC were summarized using mean and standard deviation. An independent *t*-test was used to determine mean differences between means of categorical variables with two categories while an ANOVA was used for those with three categories. A *p* value of 0.05 was interpreted as significant.

## Abbreviations

DM: diabetes mellitus
EDTA: ethylenediaminetetraacetate
eGFR: estimated glomerular filtration rate
EPO: erythropoietin
FBG: fasting blood glucose
Hb: hemoglobin
HbA1c: glycated hemoglobin
IL-6: interleukin-6
MCV: mean cell volume
PCV: packed cell volume
TIBC: total iron-binding capacity
WHO: World Health Organization
